# Effects of *Tribulus terrestris* L. on Sport and Health Biomarkers in Physically Active Adult Males: A Systematic Review

**DOI:** 10.3390/ijerph19159533

**Published:** 2022-08-03

**Authors:** Diego Fernández-Lázaro, Cesar I. Fernandez-Lazaro, Jesús Seco-Calvo, Evelina Garrosa, David P. Adams, Juan Mielgo-Ayuso

**Affiliations:** 1 Department of Cellular Biology, Genetics, Histology and Pharmacology, Faculty of Health Sciences, University of Valladolid, Campus of Soria, 42003 Soria, Spain; 2 Neurobiology Research Group, Faculty of Medicine, University of Valladolid, 47005 Valladolid, Spain; 3 Department of Preventive Medicine and Public Health, School of Medicine, University of Navarra, Navarra Institute for Health Research (IdiSNA), 31008 Pamplona, Spain; 4 Institute of Biomedicine (IBIOMED), Physiotherapy Department, University of Leon, Campus de Vegazana, 24071 Leon, Spain; dr.seco.jesus@gmail.com; 5 Psychology Department, Faculty of Medicine, Basque Country University, 48900 Leioa, Spain; 6 Department of Cellular Biology, Genetics, Histology and Pharmacology, Faculty of Medicine, and Institute of Neurosciences of Castile and Leon (INCYL), University of Valladolid, 47005 Valladolid, Spain; evelinags17@gmail.com; 7 Dual Credit Enrollment Program, Point University, Savannah, GA 31419, USA; david.adams@point.edu; 8 Department of Health Sciences, Faculty of Health Sciences, University of Burgos, 09001 Burgos, Spain; jfmielgo@ubu.es

**Keywords:** *Tribulus terrestris*, sport supplement, biomarkers, physically active adults, systematic review

## Abstract

*Tribulus terrestris* L. (*TT*) is a plant used in traditional Chinese medicine, Ayurvedic medicine, and sports nutrition to improve health and performance. However, no conclusive evidence exists about the potential beneficial effects of *TT* on sport and health biomarkers in physically active adults. Based on the Preferred Reporting Items for Systematic Reviews and Meta-Analyses (PRISMA) guidelines, and the modified McMaster Critical Review Form for methodological quality assessment, we systematically reviewed studies indexed in Web of Science, Scopus, and PubMed, to assess the effects of *TT* on immunological, hematological, biochemical, renal, lipidic, hormonal behavior, and anti-inflammatory response in physically active adult males. Among 340 records identified in the search, a total of 7 studies met the inclusion and exclusion criteria. Overall, participants supplemented with *TT* displayed significant improvements in lipid profile. Inflammatory and hematological biomarkers showed moderate beneficial effects with no significant changes on renal biomarkers. No positive effects were observed on the immune system response. Additionally, no *TT*-induced toxicity was reported. In conclusion, there was no clear evidence of the beneficial effects of *TT* supplementation on muscle damage markers and hormonal behavior. More studies are needed to confirm the benefits of *TT* due to the limited number of studies available in the current literature.

## 1. Introduction

Sports nutrition influences athletes’ health and sports performance [1]. When the nutritional recommendations are not sufficient to meet athletes’ requirements, athletes resort to using supplements to improve their athletic performance [2]. Dietary supplements are intended to cover the specific nutritional needs of physically active populations that may vary depending on the intensity and duration of exercise, sport discipline, time of the season, age, physical condition, and external environment [3]. Herbal supplements contain active botanical phytochemicals (phenolic acids, alkaloids, flavonoids, glycosides, saponins, and lignans) extracted from seeds, gums, roots, leaves, bark, berries, or flowers, of the plants [4]. Sports supplements may additional help to maintain athletes health and improve and maximize their athletic performance [5].

Ergo nutritional supplement sales have annually increased in the U.S. market, and it is estimated an annual growing rate of 8.9% over the next years [6]. One type of dietary supplements used in exercise and sports are herbal supplements [7], which are ranked second in sales in the U.S. dietary supplement market, only behind vitamin supplements [6]. Among herbal supplements, an extract of an exotic plant called *Tribulus terrestris* L. (*TT*) [7], has increased popularity among consumers.

*TT* is an herbaceous plant originally from India that belongs to the *Zygophyllaceae* family that comprises about 20 worldwide species. *TT* is known variously as “*Tribulus*”, “puncture vine”, “caltrop”, “goat head”, “puncture weed”, “Mexican sandbur”, “Texas sandbur” or “bullhead”, among other names. *TT* predominantly grows in the countries around the Mediterranean Sea and in sub-tropical regions around the world [8]. *TT* is a popular in traditional Chinese medicine and it has been widely used in many countries for thousands of years. *TT* (fruits and roost) has been mainly used for its potential cardioprotective, antiurolithic, antidiabetic, anti-inflammatory, antitumor, and antioxidant effects [9]. *TT* is composed of saponins, polyphenols, flavonoids, glycosides, alkaloids, and tannins [8]. Steroidal compounds, such as saponins, are heterosides consisting of a glycoside and a genin part, which can be steroidal or triterpene in nature. Steroid genins are derived from a hexacyclic skeleton of 27 carbon atoms, which is the spirostane nucleus [8,10]. Saponins are responsible for the positive physiological effects of *TT* on sexual performance, coronary heart disease, avoidance of ischemia/reperfusion injury to the heart, and modulation of hypertension [8,10].

Currently, products with *TT* extracts are commonly used by numerous athletes and non-athletes to improve testosterone levels and strength performance [11]. *TT* is attractive for athletes because of its potential ergogenic effects on sports performance [12], improving recovery after exercise [13], strength, and stimulating skeletal muscle hypertrophy [14] associated with *TT* as a testosterone booster [11], and promoting muscle anabolism [10,13]. These effects have been suggested to be linked to the saponins contained in *TT* [15]. The saponins of *TT* could exert an anabolic/androgenic influence by activating endogenous production of testosterone and luteinizing hormone (LH), which may stimulate muscle growth (7,8). The polyphenols and flavonoids contained in *TT* act as antioxidants because they effectively scavenge free radicals in a concentration-dependent manner [8,10]. Moreover, *TT* may reduce inflammation [16] by attenuating muscle damage [17] and oxidative damage [18].

Blood circulation is responsible for biochemical, metabolic, hormonal, and immunological processes, and for maintaining homeostasis in the body. Therefore, it is important to assess the physiological changes and adverse effects that may occur under the influence of sports activity or nutritional supplements used through biomarker analysis [19]. Additionally, knowledge of the physiological action of supplements for physicians, nutritionists, coaches, and athletes in the sports field may be of interest considering many new over-the-counter herbal products, with little evidence given the limited number of clinical trials and updated information. To date, there are scarce studies on the effects of *TT* on sports biomarkers in healthy physically active adults. Biomarker monitoring may provide crucial information to adjust exercise workloads to maintain an optimal level of health. Thus, the purpose of this study was to systematically review current studies on the potential effects of *TT* supplementation on immunological, hematological, and biochemical biomarkers, hormonal behavior, lipid profile, renal function, and anti-inflammatory activity in physically active healthy adult males.

We used the PICO model according to the standard methods proposed by the Preferred Reporting Items for Systematic Reviews and Meta-Analyses Guidelines (PRISMA) [20] as follows: *Population*: physically active healthy adult males; *Intervention*: supplementation with *TT*; *Comparison*: placebo/control group or pre/post comparison data group; *Outcomes*: immunological (white blood cells [WBC], lymphocytes [LYM], monocytes [MON], and granulocytes [GRAN]); hematological (hemoglobin [HB], hematocrit [HCT], and red blood cells [RCB]); biochemical (creatine kinase [CK], lactate dehydrogenase [LDH], and bilirubin [BIL]); hormonal (testosterone, dihydrotestosterone [DHT], estradiol [E2], luteinizing hormone [LH], insulin-like growth factor [IGF-1], insulin-like growth factor binding protein [IGFBP-3], growth hormone [GH], cortisol, ratio testosterone/cortisol, and ratio testosterone/epitestosterone); lipidic (cholesterol [Chol], triglycerides [Tg], low-density lipoprotein [LDH-Chol], high-density lipoprotein [HDL-Chol]); renal (creatinine [Cr], uric acid [Ua]; blood urine nitrogen [BUN], urea); inflammatory (interleukin 6 [IL-6], high-sensitivity C-reactive protein [Hs-CRP], erythrocyte sedimentation rate [ESR]) biomarkers.

## 2. Materials and Methods

### 2.1. Search Strategy

We established a structured search via the databases Scopus, Web of Science (WOS), and Medline (PubMed) for studies published from database inception to 29 April 2022. The search strategy contained a mix of Medical Subject Headings (MeSH) and free words for key concepts related that included: (“Tribulus” OR “puncture vine” OR “caltrop” OR “goat head”, “puncture weed” OR “Mexican sandbur” OR “Texas sandbur” OR “bullhead”) AND (“Immunological” OR “Biochemical” OR “Hematological” OR “Hormonal” OR “ Lipid profile” OR “Inflammatory”) AND (“Effects” OR “Biomarkers” OR “Parameters” OR “Bioindicators” OR “Biological Activity” OR “Pathways”). The search for published studies was independently performed by 2 authors (D.F.L. and J.M.A.) and disagreements about records were resolved by a third reviewer (J.S.-C.)

### 2.2. Selection Criteria

The following inclusion criteria were applied to select studies: (a) original records with randomized and non-randomized trials, double-blind or parallel controlled design; (b) records that evaluated the impact of *TT* supplementation administered alone in physically active adult males; (c) records with specific information on the dose of *TT* intake and intervention period; (d) studies with information about the type of pharmaceutical form used for the supplementation (pills, tablets, gel caps, liquids); (e) records that examined at least one reported outcomes related to any of the hematological, inflammatory, antioxidant, and biochemical biomarkers before communicated. On the other hand, the exclusion criteria were the following: (a) studies conducted on animals, and/or in vitro studies; (b) studies in which *TT* was administered with other supplements or administered as a combination; (c) records in which the outcomes were not related to sports performance or health; (d) editorials, reviews, notes, and any other non-original studies; (e) studies conducted in participants with any cardiovascular, metabolic, musculoskeletal, or other chronic disorder.

### 2.3. Quality Assessment

The methodological quality assessment of the selected records was performed using the McMaster University Occupational Therapy Evidence-Based Practice Research Group [21]. The aim of this evaluation was to exclude studies with poor methodology.

### 2.4. Data Extraction

The following information was extracted from each study included in the systematic review: included name of the first author; publication year; country where the study was conducted; study design; sample size; participants’ sex and age; weight or body mass index (BMI); dosage, i.e., specific amount, number, frequency, and percentage of saponins), timing of the supplementation; duration of intervention; outcomes reported; and final results. Two investigators (D.F.-L. and J.M.A.) conducted the data extraction process using a spreadsheet. In case of disagreements related to the data extraction, a third author reviewer (J.S.-C.) was involved in the process.

## 3. Results

### 3.1. Study Selection

**Figure 1:**
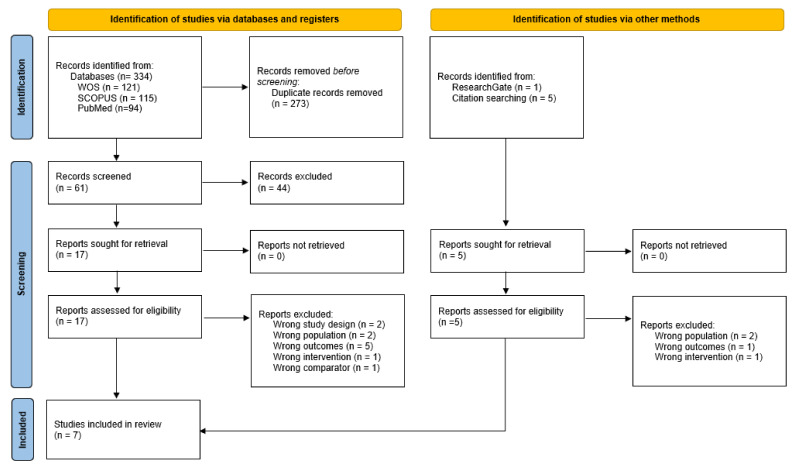
Flow diagram depicting the identification and selection processes of relevant studies according to Preferred Reporting Items for Systematic Reviews and Meta-Analyses (PRISMA) guidelines.

A total of 340 studies were identified, 334 studies were from 3 electronic databases WOS, SCOPUS, and PubMed, and 6 studies were retrieved from reference lists of selected electronic databases studies. After exclusion of 273 duplicates, a total of 61 articles identified in databases and registries were examined. After evaluation of the title and abstract, 17 articles were considered as potential registries. After review of the full text and evaluation of potential records from databases and registries as well as other sources, 7 [12,14,15,17,22,23,24] studies were included in the systematic review (Figure 1).

### 3.2. Quality Assessment

Four studies [12,14,15,17] were considered as “excellent quality” and 3 [22,23,24] as “very good quality” (Table 1).

### 3.3. Characteristics of the Participants and Interventions

The characteristics of the participants are shown in Table 2. The total number of volunteers was 165 men, all of them healthy (without any chronic conditions that prevented the practice of physical activity) and physically active. Three studies [12,14,23] included elite athletes who practiced basketball [23], rugby [14] and boxing [12]. Two studies [15,22] included highly trained athletes with at least 20 months of CrossFit® [15] and endurance sports experience within the study program of physical education [22]. Two studies [17,24] did not include participants with regular physical activity habits prior to baseline. However, in these studies participants performed either 4 sessions of scheduled physical activity (aerobic and anaerobic) [24] or were instructed to maintain routine daily physical activity [17] during the length of the study.

*TT* supplementation was used in 5 studies [12,14,15,22,23] as commercially registered product and in 2 studies [17,24] was specifically prepared for the intervention. Doses of *TT* supplementation varied from 1875 mg [22] to 450 [14] mg, with 2 studies that supplemented with 2 doses, 1800 mg or 2700 mg [23], and 900 mg or 1800 mg [24]. Supplementation duration varied from 12 weeks [24] to 20 days [22]. Authors supplemented participants after breakfast and before going to sleep [23], in the morning and before going to sleep [24], in the morning [12] or in the morning and afternoon [17,22]. Two studies did not report dose schedule [14,15]. No adverse effects were reported, and, in general, the participants tolerated the *TT* supplementation well [12,14,15,17,22,23,24].

### 3.4. Outcome Evaluation

Table 3 summarizes the contents of the studies contained in this systematic review.

#### 3.4.1. Immunological Biomarkers

One study [22] analyzed the impact of *TT* on immunological markers. In LYM, MON, and GRAN, no significant changes were observed when comparing the intervention group with respect to the control group. However, a significant increase in GRAN and a significant decrease in LYM were observed after 20 days of *TT* supplementation compared to baseline levels.

#### 3.4.2. Hematological Biomarkers

Hematological biomarkers were evaluated by Milasius et al. [22]. This study observed a non-significant increase on HCT and HB, however, RCB and MCV showed a downward trend when comparing the *TT* supplementation group with the control group. Only HCT levels showed a non-significant increase from baseline to the end of training in the *TT* group [22].

#### 3.4.3. Biochemical Biomarkers

The effect of *TT* on CK was evaluated in 3 studies [12,17,22]. Ma et al. [12] showed that CK in the intervention group following high-intensity training was significantly lower than in the control group and from baseline to the end of the supplementation. However, Milasius et al. [22] described contrary results for athletes in endurance sports. In addition, in 1 of these studies [17], post-exercise values of this CK in the *TT* group were moderately lower compared to its pre-exercise levels. Moreover, differences between the groups regarding post-exercise CK were not significant [17].

LDH level in the *TT* group was significantly lower than in the placebo group in response to resistance exercise training [17]. BIL concentration showed a decreasing trend when comparing the *TT* supplementation group with the control group and when examining changes from baseline [22].

#### 3.4.4. Renal Biomarkers

The impact of *TT* on renal biomarkers, Cr, Ua, Ure, and BUN were evaluated in 2 studies [12,22], observing no significant changes.

#### 3.4.5. Lipid Biomarkers

Chol and LDH-Chol levels were significantly reduced and HDL-Chol were significantly increased among participants in the *TT* intervention group compared with the control group [24] and investigators also observed Chol and LDH-Chol levels were significantly reduced and HDL-Chol have significantly increased changes from baseline to the end of the intervention [24]. Moreover, Milasius et al. [22] reported a non-significant improvement in Tg over the control group and from baseline to the end of supplementation.

#### 3.4.6. Inflammatory Biomarkers

Inflammatory biomarker studies were examined in 2 studies [17,22]. IL-6 and Hs-CRP showed a downward trend when comparing the *TT* supplementation group with the control group [17], however, examining changes from baseline observed significant increases in IL-6 and Hs-CRP [17]. One study [22] reported changes in ESR during the study, but non-statistical significance.

#### 3.4.7. Hormonal Biomarkers

Testosterone levels were examined in 5 studies [12,15,22,23,24]. Two studies reported significant increases in testosterone levels in the *TT* intervention group relative to the control [15,24]. Examining changes from baseline, 2 studies showed a trend improvement [12,22] and other investigators [24] observed significant increases in testosterone levels. However, the study by Poprzecki et al. [23] reported significant decreases in testosterone levels. Regarding cortisol levels, 2 studies showed contradictory findings when comparing the *TT* supplementation groups from baseline [15,22]. IGF-1 and GH observed significantly increased levels from baseline to the end of the intervention, and also between the intervention and control group [24]. However, Ma et al. [12] showed a decreasing trend in all experimental situations for IGF-1 and DHT.

## 4. Discussion

The purpose of this systematic review was to critically evaluate the effects of *TT* supplementation on sports biomarkers in physically active healthy adult males. A total of 7 studies met the inclusion/exclusion criteria. Participants supplemented with *TT* presented significant improvements in lipid profile. Inflammatory and hematological biomarkers showed moderate beneficial effects with no significant changes on renal biomarkers. However, *TT* seemed not to have a positive effect on the immune system. There was no clear evidence of the beneficial effects of *TT* supplementation on muscle damage markers and hormonal behavior. No *TT*-induced toxicity was reported.

### 4.1. Tribulus terrestris L. Supplementation

Supplementation doses administered in interventions varied from 2700 [23] to 450 mg [14], from 20 days [22] to 3 months [24]. None of the 165 men included in this systematic review suffered adverse effects during the *TT* supplementation protocol. No significant abnormal changes in renal function biomarkers (Cr, Ua, Urea, and BUN) were found [12,22], and a downward trend on BIL levels was observed [22]. Therefore, no drug-induced nephrotoxicity or hepatotoxicity was reported. This is not consistent with other human clinical studies that have reported gastrointestinal problems, such as stomach pain or gastric reflux [25,26], gynecomastia [27], priapism [28], nephrotoxicity [29,30], hyperbilirubinemia [29], hepatotoxicity, and neurotoxicity [30] when taking *TT.* Sleep disturbances, exhaustion and fatigue, hypertension, and elevated heart rate have been reported after administration of *TT* at doses ≥1000 mg per day [10,29]. In rats, toxic effects on liver and kidney have been observed, and an in vitro study has indicated cytotoxicity and/or genotoxic activity [31] and nephrotoxicity [32]. The report of the *Scientific Committee of the Spanish Agency for Consumer Affairs, Food Safety and Nutrition* (AECOSAN) reported that the recommended daily intake of *TT* would be between 250 and 9000 mg [31]. *TT* has not been listed as a banned substance by the *World Anti-Doping Agency* (WADA) [33]. However, *TT* may increase the testosterone/epitestosterone ratio in urine above the WADA permitted limits (4:1) [14], and may cause athletes to inadvertently test positive for testosterone [33]. There are some sports agencies such as the Australian Institute of Sport, the National Centre for Sports Medicine in Poland and the Medical Commission of the Polish Olympic Committee, and the Canadian Cycling Association that may issue a positive test result in a doping control by *TT* supplementation [9].

### 4.2. Hematological Biomarkers

The study by Milasius et al. [22] included in this systematic review, showed no abnormal changes in hematological biomarkers during 1875 mg per day *TT* supplementation containing 100% saponins. Investigators observed a moderate increase of HCT and HB compared to control group, but did not have positive effect on RCB and MVC. *TT* does not directly affect erythropoietic activity in persons. This property may be explained because *TT* supplementation showed a trend in improvement of testosterone levels [22]. Testosterone stimulates erythropoiesis, induces erythrocytosis, and increases HCT levels in a dose-dependent manner without an associated increase in erythropoietin levels [34]. Another pathway may be through the action of DHT, Protodioscin (5,6-dihydroprotodioscin, neoprotodioscin), which is believed to increase the conversion of testosterone to DHT, which promotes red blood cell production [10]. Physical activity causes alterations in different hematological parameters. Thus, the adequate hematological status in the organism is an essential factor that conditions physical capacity, sports performance, and health in athletes. Therefore, those factors that improve the transport and utilization of oxygen at the muscular level [1]. Thus, steroidal saponins from *TT*, like Protodioscin, could have beneficial effects on physical fitness [9], by improvements on HB and HCT, and other ergogenic aids such as iron supplements [3].

### 4.3. Immunological Biomarkers

Steroidal saponins contained in *TT* had demonstrated immunostimulant activity on macrophage activity in vitro assays and activation of non-specific immunity in animal model [35]. In addition, *TT* showed an enhancement of B cell activity with significant increases on serum antibody titers that are potentially effectors of the humoral response [36]. These findings are contradictory to those described by Milasius et al. [22], where these investigators asserted a negative effect on the immune system. Milasius et al. [22] have reported that 1875 mg of *TT* for 20 days induces a change in leukogram transformed into granulocytes with substantial decrease in lymphocytes and significant increase of neutrophils, basophils, and eosinophiles in endurance athletes. In this regard, dose and/or the high percentage of steroid saponins in *TT* supplementation could be responsible for the immunosuppression, which could be like that of corticosteroids [37].

However, it should be considered that intense and acute physical exercise is accompanied by responses that are remarkably similar in many respects to those induced by infection, sepsis, or trauma. The number of circulating leukocytes (mainly lymphocytes, monocytes, and neutrophils) and their magnitude are related to both the intensity and duration of exercise [38]. For this reason, it could be hypothesized that the effects on the cells of the immune system are not due to *TT*-induced toxicity, but because *TT* is not able to restore the effects that exercise has on the immune system. In this sense, no immunosuppressive effect of *TT* has been reported to date [10,29]. Therefore, we recommend evaluating blood immunological biomarkers during periods of *TT* supplementation in athletes.

### 4.4. Biochemical Biomarkers

Intense and prolonged periods of physical activity increases circulating levels of LDH and CK, which negatively affect athletes by decreasing muscle performance [1]. This systematic review examined CK [12,17,22], and LDH [17], as potent muscle damage biomarkers. In these studies, *TT* supplementation demonstrated a significant reduction for CK activity (during high-intensity training) [12] and LDH activity (during resistance-exercise training) [17]. Furthermore, in 1 of these studies [17], post-exercise CK activity was moderately lower compared to its pre-exercise levels. These results are consistent with previous animal study [39]. Potentially, the decrease in CK or LDH after *TT* supplementation could be attributed to an antioxidant role [8]. In this sense, the protective effect appears to be mediated directly either through inhibition of through inhibition of tissue peroxidation [40] or induced increase serum superoxide dismutase (SOD) activity [41]. Previously, the anti-inflammatory effect of *TT* has been reported by its inhibitory action on cyclooxygenase-2 (COX-2) expression [42]. This property could influence the reduction of CK activity. The action of *TT* could markedly reduce histamine and/or prostaglandin production by its inhibitory action on COX-2 [2]. Consequently, in local areas of inflamed skeletal muscle, membrane permeability would be reduced, thereby reducing the intracellular-intravascular flux of CK. However, Milasius et al. [22] found significant increases in CK activity. These discrepancies may be due to the composition of the *TT* supplement administered. Perhaps, several bioactive components of *TT* such as flavonoids, alkaloids, phenols, and saponins [12,17] are necessary to have an antioxidant and/or anti-inflammatory effect, and not only steroidal saponins [22].

Another blood biomarker included in this study is BIL [22]. In athletes, BIL elevations are frequent and derive from repeated micro-trauma that breaks down HB and metabolizes into BIL, demanding strength training or high levels of physical and/or mental stress due to sports competitions [43]. Moreover, Milasius et al. [22], have observed a downward trend in the concentration of BIL, liver function biomarker. *TT* showed a remarkable hepatoprotective activity, that seems to be related to inhibition of lipid peroxidation and increased levels of antioxidant enzymes, in addition to free radical scavenging action [44,45]. *TT*’s flavonoids would enhance the viability and cellular leakage of transaminases (AST, ALT) and could be responsible for protecting the liver against oxidative damage and tissue-damaging enzymatic activities. However, we must also consider that *TT* plant contains alkaloids, steroidal glycosides, and steroidal saponins [46].

### 4.5. Inflammatory Biomarkers

It is known that exercise induces an increase in plasma levels of IL-6, which may be the indicator of a more intense inflammatory response [38]. Increased IL-6 precedes inflammatory cytokines such as TNF-α, macrophage inflammatory protein-1 (MIP), IL-4, IL-1, and acute phase proteins such as CRP [43]. This would have negative repercussions on the athlete’s muscular system due to increased inflammation or leukocyte infiltration. *TT* extracts are known to have anti-inflammatory activities in in vivo and in vitro assays [46]. Additionally, *TT ’s* properties would include down-regulation of enzymes responsible for the production of cytokines and inflammatory mediators [47]. These findings were in agreement with the only study that evaluated the anti-inflammatory effect of *TT*, then 500 mg × day^−1^ for 2 weeks of *TT* supplementation was sufficient to induce a moderate but not statistically non-significant decrease in IL-6 and Hs-CRP [17]. A plausible pathway would be that *TT* inhibits NF-κB a signaling pathways [48]; this property is like that of another herbal supplement, curcumin [2]. Down-regulation of NF-κB [48], plus inhibition of COX-2 [42], allows *TT* to suppress leukocyte infiltration, activation, and maturation, as well as the production of proinflammatory mediators TNF-α and IL-4, at the focus of inflammation. Control of IL-6 levels could attenuate a wide range of inflammatory events that affect the homeostasis of the organism. These properties are similar to other nutritional supplements such as glycophosphopeptical AM3 [37]. ESR is an indirect measure of the degree of inflammation present in the body [43]. One study included in this systematic review [22] showed modest increases in ESR in *TT* group. Maybe this is because the anti-inflammatory effect requires several active ingredients of *TT* and saponins alone would not be sufficient.

Overall, *TT*, as a nutraceutical, has been used in traditional Chinese medicine, in Ayurvedic medicine in India, as well as by modern herbalists as an adjuvant for its various anti-inflammatory properties in inflammation-mediated diseases such as diabetes, obesity, pancreatitis, cancer, inflammatory bowel disease, kidney disease, and arthritis [49].

### 4.6. Renal Biomarkers

Cytoprotective power on the cells of the renal system would suggest improvements in renal function after *TT*’s treatment [8], but 2 studies included in this systematic review did not significantly change after *TT* supplementation in renal biomarkers [12,22]. All of them were in physiological range values, suggesting that there was no *TT*-induced renal toxicity. These results agree with those reported by Gandhi et al. [32] who neither observe improvements in renal function nor found *TT*-induced toxicity. However, kidney injury after *TT* supplementation has been described [29,30].

Concerning the Cr, Ua, Urea, and BUN (high-intensity training) levels, athletes of the studies integrated in this systematic review did non-significantly decrease when compared with baseline (*TT* group) [12,22]. BUN (high-volume training) levels increased from baseline but were not observed when compared with control groups [22]. The concentration of BUN remained within normal range (7–21 mg/dL) and most likely reflected muscle destruction increments due to high-volume training [22]. In this line, studies in rats [50] and renal epithelial cell lines (NRK-52E) [51], *TT* showed significant dose-dependent protection against elevation of biochemical parameters in urine. This premise would add new perspective of *TT* on the maintenance in physiological range of renal biomarkers that are increased after exercise.

### 4.7. Lipid Biomarkers

Moderate physical exercise is associated with a healthy plasma lipid profile and a lower risk of coronary artery disease and cardiovascular mortality [52]. Physical exercise increases plasma HDL-Chol values and decreases LDL-Chol and Chol [53,54]. However, stressful physical exercise can increase cardiovascular risk [55]. Direct and significant associations have been found with the degree of physical stress to which the different athletes were subjected. The most common alterations in lipid biomarkers were on HDL-Chol and LDL-Chol [55,56].

*TT* has demonstrated lipid-lowering activity [8]. *TT* supplementation in animal models leads to a decrease in Chol and LDL-Chol, and an increase in HDL-Chol levels in the blood [57,58]. Additionally, the lipid-lowering preventive effect of *TT* was demonstrated on diet-induced hyperlipidemia in mice by the decrease in Chol and LDL-Chol blood levels [59]. These results are consistent with 3 studies included in the review in which *TT* has shown lipid-lowering activity [17,22,24]. Phenolic compounds seem to be responsible for the direct effects and saponins for the preventive effects on the lipid profile [46]. Phenolic compounds may be increased lipase activity in skeletal muscle and decreased activity in adipose tissues which stimulate the use of plasma triglycerides as energy fuel by muscle and blocks their storage as fat [57]. Saponins would increase the activity of SOD in the liver, improving the antioxidant capacity [59]. These results support the use of *TT* as a sport supplement to counterbalance and equilibrate lipid profile to create lipid level balances in situations of high physical stress or demanding exercise.

### 4.8. Hormonal Biomarkers

Intensive exercise has been shown to induce a dysfunction of the hypothalamic-pituitary-testicular axis, especially testicular impairment by causing a suppression of testosterone secretion during the latter stages of exercise. Additionally, during intense prolonged exercise, Adrenocorticotropic Hormone (ACTH) concentrations increase, resulting in a significant release of cortisol [1]. These alterations on hormonal behavior would cause sports performance to decrease [5]. *TT* has become popular for its anabolic properties that could potentially raise the blood testosterone level and stimulate hypertrophy of the skeletal muscles of Bulgarian weightlifters [11].

Investigators in 4 studies reported beneficial effects of *TT* supplementation on testosterone levels [12,15,22,24]. These results are consistent with animal studies [13,60,61,62] or older men [63] in which *TT* has shown to increase testosterone. The properties of *TT* may also influence the effects on testosterone levels because of their pleiotropic effects such as: (i) direct action of the pituitary gland that secretes more LH; (ii) increased levels of dehydroepiandrosterone (DHEA) molecules; (iii) suppression of aromatase that prevents estrogen synthesis; (iv) antioxidant effect that protects against endothelial dysfunction in the gonads; (v) anti-inflammatory properties [8,46,64]. *TT*’s steroidal saponins, such as gitonin, protodioscin, and tribulosaponins A and B, would be responsible for stimulating testosterone production by these multiple pathways [46]. Nonetheless, in the study of Poprzecki et al. [23], included in the present review, the levels of testosterone when supplementing with *TT* significantly decreased. Moreover, *TT* provided no benefit on plasma testosterone levels in rugby players, with a marked decrease in the testosterone/epitestosterone ratio [14]. Likewise, other clinical trials reposted that *TT* was no more effective than placebo for serum total testosterone concentration [65]. The testosterone-enhancing direct actions of *TT* have been ratified by some investigators, as shown above, but are questioned by other studies.

The indirect hormonal behavior of testosterone showed contradictory results. Poprzecki et al. [23] showed a remarkable increases in LH, due to the direct action of steroid saponins on the pituitary gland which secretes more LH [46]. LH increases could stimulate testosterone levels due to indirect action where LH regulates the expression of 17β-hydroxysteroid dehydrogenase, which is the enzyme that converts androstenedione to testosterone [46]. However, no testosterone increases are observed after 4 weeks of *TT* supplementation [23]. Increases in E2 could decrease short-term testosterone levels by aromatase activity. Although, it is difficult to explain these elevated E2 levels by the anti-aromatase activity of the bioactive components of *TT* [8,11]. Perhaps the intensity and high levels of demand of professional basketball outweigh the effects of *TT* on hypothalamic-pituitary-testicular axis.

DHT is an active androgen of testosterone. Saponins, mainly protodioscin, induce the transformation of testosterone into DHT, through their intervention on the enzyme 5-α reductase [46]. Previously, significant increases on testosterone and DHT levels have been described in primates after *TT* administration compared to control [61]. However, Ma et al. [12] reported decreases in *TT* supplementation group in both testosterone and DHT hormones. These contradictory findings may be explained by the different percentage of steroidal saponins, the dose or the bioavailability of *TT*, which were higher in the animal study [61].

Two studies included in this review evaluated cortisol levels, showing opposite results. Milasius et al. [22], in endurance athletes, showed an elevation of cortisol from baseline but in the study conducted by Fernández-Lázaro et al. [15], in CrossFit^®^ athletes, the results showed a decrease in cortisol from baseline and when compared to the control group. In addition, these investigators [15] described remarkable improvements in the testosterone/cortisol ratio which indicates adequate fatigue control may have mitigating effects on the hypothalamic-pituitary-adrenal (HPA) axis activity by cortisol-level reduction in athletes. This difference in the results may be influenced by the characteristics of the athletes in each group, the type of exercise performed or the supplementation strategy.

These results showed uncertainty about the use of *TT* as an anabolic sports supplement to counteract and balance biomarker levels to create balances at the hormonal level.

Athletes may prefer to use *TT* supplementation rather than other supplements due to its potential anabolic effects, uncommon side effects [15], and because *TT* is not listed as a banned substance by the WADA [33]. IGF-1 is a polypeptide hormone secreted by multiple tissues in response to GH. IGF-1 and GH have a great effect on muscle hypertrophy, muscle repair, alleviation of muscle damage, changes in body composition, and improvement of athletic performance. Downward alterations in GH and IGF-1 levels have been shown as a function of subject characteristics, physical activity (type, intensity, and duration), and training status [66]. A 12-week supplementation of *TT* showed significant improvements in blood concentration of GH and IGF-1 [24]. These results indicate that *TT* treatment may be a successful form of skeletal muscle protection and in accelerating skeletal muscle regeneration/repair. In opposition, supplementation with 1250 mg of *TT* extracts decreased the plasma level of IGFBP-3 and IGF-1 in male boxers after both types of training (volume/intensity) [12]. Nevertheless, it significantly alleviated muscle damage and promoted athletic performance. It is hypothesized that these findings may be mediated by the decrease in IGFBP-3, and late increasing IGF-1 bioactivity [12]. Although different results have been reported different effects of *TT* on IGF-1 and GH, the goal of supplementation is met by improving athletic performance and muscle condition.

Of particular interest for middle-aged adults are the results obtained in the study of Wilk et al. [24] on a population aged 45–60 years. In this study [24] significant improvements in hormonal behavior that could mitigate the effects of sarcopenia were observed. Sarcopenia includes an involution of skeletal muscle that is accentuated after the age of 40 years with losses in muscle strength, physical performance, and quantity/quality of skeletal muscle mass [67]. Restoration of hormonal homeostasis is one of the potential therapies to control sarcopenia [68]. Therefore, *TT* could restore and modulate the hormonal levels diminished by age.

There are many published reviews on the phytochemical and/or pharmacological profile of *TT* [46,64], however, this systematic review and reports concern the effects on sports biomarkers and health in healthy, physically active adults. So, this study could provide additional relevant information to the knowledge of *TT*. Regular physical exercise and sports supplementation induce physiological and metabolic adaptations, which influence sports performance [1]. To monitor these adaptations, blood biomarkers have been proposed as suitable markers to measure the effect of short- and long-term training/supplementation, but also to maintain health, identify chronic stress, inflammation, fatigue or as injury prevention [69]. Therefore, health and sports performance monitoring in regular exercisers would include biochemical, immunological, inflammatory, hormonal, and hematological parameters to ensure a correct state of health and the absence of any abnormality that may decrease exercise performance.

### 4.9. Limitations and Stregths

Several limitations need to be acknowledged. First, the systematic review included a limited number of records. Moreover, most of these studies included a small sample size and only male participants. Second, the intervention characteristics of the studies such intensity and duration of exercise, timing and dose of *TT* supplementation widely varied across studies. Despite these limitations, the strengths of this systematic review rely in the use of the PRISMA guidelines [20] and the McMaster Quantitative Review Form [21], the use of three electronic databases, the addition of grey studies to the search, and the inclusion of multiple outcomes generally described in herbal medicine to assess sports performance and health status.

## 5. Conclusions

The evidence presented in the studies in this systematic review showed that *TT* supplementation is safe although side effects have been reported in other animal and human studies. Given the improvements in certain biomarkers, it may also benefit physically active adults. The pleiotropic effect of *TT* may act to counteract and modulate certain physiological biomarkers out of the normal range by intense and stressful physical exercise. In this regard, some examples are hematological, lipid, renal, and hepatic biomarkers. There are doubts about the potential effect on muscle damage, anti-inflammatory effect, and hormonal biomarkers. In addition, it lacks effect on the immune system. However, more research is needed to confirm the potential health benefits of *TT* supplementation in healthy adults without chronic diseases.

## Figures and Tables

**Table 1:** Results of the methodological quality assessment of included studies—McMaster Critical Review Form for Quantitative Studies [21].

Study	Item																Total	%	Quality Score
	1	2	3	4	5	6	7	8	9	10	11	12	13	14	15	16			
Ma et al., 2017 [12]	1	1	1	1	0	1	1	1	1	0	1	1	1	1	1	1	15	93.8	E
Rogerson et al., 2007 [14]	1	1	1	1	0	1	1	1	1	1	1	1	1	1	1	1	15	93.8	E
Fernández-Lázaro et al., 2021 [15]	1	1	1	1	1	1	1	1	1	1	1	1	1	1	1	1	16	100	E
Talemi et al., 2021 [17]	1	1	1	1	1	1	1	1	1	1	1	1	1	1	1	1	16	100	E
Milasius, et al., 2019 [22]	1	1	1	1	0	1	1	0	1	1	1	0	1	1	1	1	13	86.7	VG
Poprzecki et al., 2005 [23]	1	1	1	1	0	1	1	1	0	0	1	1	1	1	1	1	13	86.7	VG
Wilk et al., 2012 [24]	1	1	1	1	0	1	1	0	0	1	1	1	1	1	1	1	13	86.7	VG

Abbreviations: 0 = not fulfilled criterion; 1 = fulfilled criterion; E = excellent; VG = very good. Item 1: study purpose; item 2: literature review; item 3: study design; item 4: blinding; item 5: sample description; item 6: sample size; item 7: ethics and consent; item 8: validity of outcomes; item 9: reliability of outcomes; item 10: intervention description; item 11: statistical significance; item 12: statistical analysis; item 13: clinical importance; item 14: conclusions; item 15: clinical implications; item 16: study limitations.

**Table 2:** Characteristics of participants and supplementation protocols of the selected studies.

Characteristics	Types	Study
Participants	Elite athletes	[12,14,23]
	Well-trained athletes	[15,22]
	No regular training before the study	[17,24]
Supplementation product	Manufactured	[17,24]
	Registered product^®^	[12,14,15,22,23]
% Saponins of the supplementation product	100%	[22,23,24]
	60%	[14]
	>40%	[15,17]
	40%	[12]
Total dose (mg) × day^−1^	1875	[22]
	1800–2700	[23]
	1250	[12]
	900–1800	[24]
	770	[15]
	500	[17]
	450	[14]
Duration	12 weeks	[24]
	6 weeks	[15]
	5 weeks	[14]
	4 weeks	[23]
	3 weeks–4 weeks (rest)–3 weeks	[12]
	2 weeks	[17]
	20 days	[22]
Dose schedule	After exercise and before going to sleep	[23]
	a.m.	[12]
	a.m. and before going to sleep	[24]
	a.m. and p.m.	[17,22]
	No reported	[14,15]

Abbreviations: a.m. = ante meridiem; p.m. = post meridiem.

**Table 3:** Studies included in the systematic review of the effect of *Tribulus terrestris* L. on hematological and biochemical markers, hormonal behavior, and oxidant response in healthy adults.

First Author, Year of Publication, and Country	Study Design	Participants (Baseline Sample Size and Characteristics, Withdrawals, and Final Group Sample Size)	Intervention	Outcomes	Results	
Ma et al., 2017, China [12]	Random-ized, double-blind, placebo-controlled trial	15 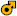 boxers (national second-level athletes, 2–3 y of training) Age (mean ± SD): CG: 16.6 ± 1.9 y IG: 16.1 ± 1.8 y Weight (mean ± SD): CG: 62.8 ± 15.2 kg IG: 64.1 ± 6.6 kg Body fat (mean ± SD): CG: 9.6 ± 3.2% IG: 9.8 ± 2.4% 2 withdrawals/lost to follow-up 13 participants completed the study 7 participants CG 6 participants IG	2 × 625 mg *TT* (Pronova Biocare, Sweden) (>40% steroidal saponins) *“Placebo”* (starch) 2 cap every morning 3 wk supplementation For high-volume training 4 wk rest 3 wk supplementation For high-intensity training	BUN CK DHT T IGF-1 IGFBP-3	IG vs. CG	
					*high-volume training*	*high-intensity training*
					↓BUN ↑CK ↓DHT ↓Testosterone ↓IGF-1 ↓*IGFBP-3	↓BUN ↓*CK ↓DHT ↓Testosterone ↑IGF-1 ↓*IGFBP-3
					*IG: change from baseline*	
					*high-volume training*	*high-intensity training*
					↑BUN ↑CK ↓DHT ↑Testosterone ↓IGF-1 ↓*IGFBP-3	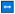 BUN ↓*CK ↓DHT ↑Testosterone ↓IGF-1 ↓*IGFBP-3
Rogerson et al., 2007, Australia [14]	Random-ized, double-blind, placebo-controlled trial	22 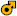 male elite rugby players Age (mean ± SD) CG: 19 ± 1.3 y IG: 20.5 ± 3.8 y Weight (mean ± SD) CG: 87.6 ± 9.0 kg IG: 88.5 ± 10.5 kg No withdrawals reported 11 participants CG 11 participants IG	450 mg/cap of *TT* extract (Body Science, Sydney, Australia) (60% steroidal saponins; 40% flavonoids, alkaloids, phenols). *“Placebo”* inert herbs identical *TT* 1 cap × day 5 wk	Testosterone/Epites-tosterone	*IG vs. CG* ↓Testosterone/Epitestosterone *IG: Change from baseline* ↓Testosterone/Epitestosterone	
Fernández-Lázaro et al., 2021, Spain [15]	Random-ized, single-blind, placebo-controlled trial	30 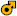 CrossFit^®^-trained athletes Age (mean ± SD): CG: 33.1 ± 5.7 y IG: 32.9 ± 6.3 y Body mass (mean ± SD) CG: 80.1 ± 10.7 kg IG: 81.2 ± 11.5 kg No withdrawals reported 15 participants CG 15 participants IG	2 caps × 385 mg *TT* (Quamtrax Europe, Spain) (40% steroidal saponins) *“Placebo”* (maltodextrin) 2 caps empty stomach 6 wk	Testosterone Cortisol Testosterone/Cortisol	*IG vs. CG* ↑*Testosterone ↓Cortisol ↑Testosterone/Cortisol *IG: Change from baseline* 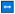 Testosterone ↓Cortisol ↑Testosterone/Cortisol	
Talemi et al., 2021, Iran [17]	Random-ized, double-blind, placebo-controlled trial	18 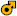 healthy physically active through resistance exercise training Age (mean ± SD) 22.44 ± 2.54 y BMI (mean ± SD) 26.15 ± 1.62 kg/m^2^ No withdrawals reported 9 participants CG 9 participants IG	2 × 250 mg *TT* *TT* powder and extract (27 mg total phenolic content, 100 mg furostanol saponins, and 34 mg total flavonoids) *“Placebo”* (maltodextrin) 2 wk daily: morning and evening intakes after meal	IL-6 Hs-CRP CK LDH	*IG vs. CG* ↓IL-6 ↓Hs-CRP ↓CK ↓*LDH *IG: Change from baseline* ↑*IL-6 ↑Hs-CRP ↓CK ↑LDH	
Milasius, et al., 2019, Lithuania [22]	Placebo-controlled study	32 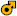 athletes’ endurance sport Age (range): 20-22 y Weight (mean ± SD): CG 76.0 ± 8.2 kg IG 75.3 ± 7.7 kg BMI (mean ± SD) CG: 22.9 ± 1.7 kg/m^2^ IG: 23.1 ± 1.9 kg/m^2^ No withdrawals reported 12 participants CG 20 participants IG	3 × 625 mg *TT* powder (Optimum Nutrition, EE.UU.) (100% furostanol saponins) a.m. (1 × cap) and p.m. (2 × caps) intakes 20 days	RCB Hb HCT MVC ERS WBC LYM MON GRAN CK Cr Ua Urea Chol Tg Bilirubin Testosterone Cortisol	*IG vs. CG* ↓RCB ↑HB ↑HCT ↓MVC ↑ERS ↓WBC ↓LYM ↑MON ↑GRAN ↑*CK ↓Cr ↑Ua ↓Urea ↓Chol ↑Tg ↓Bilirubin xTestosterone xCortisol	*IG: Change from baseline* ↓RCB ↓HB ↑HCT 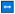 MVC ↓ERS 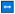 WBC ↓*LYM ↓MON ↑*GRAN ↑*CK ↓Cr ↓Ua ↓Urea ↓Chol ↑Tg ↓Bilirubin ↑Testosterone ↑Cortisol
Poprzecki et al., 2005, Poland [23]	Placebo-controlled study	24 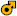 competitive basketball players Age (mean ± SD): 26 ± 3.4 y Weight (mean ± SD): 91.5 ± 9.0 kg No withdrawals reported 8 participants CG 8 participants IG	“*Tribusteron 90*” 450 mg/cap (100% Steroidal saponins) 1st 4 caps × day, 2 wk (1800 mg saponins) 2st 6 caps × day, 2 wk (2700 mg saponins) *“Placebo”* 450 mg/caps gelatin. Twice daily: 30 min before training and 20 min before going to bed 4 wk	Testosterone Luteinizing Hormone Estradiol	*IG vs. CG* ↓Testosterone ↑Luteinizing Hormone ↑Estradiol *IG: Change from baseline* ↓*Testosterone ↑Luteinizing Hormone ↑*Estradiol	
Wilk et al., 2012, Poland [24]	Random-ized, placebo-controlled trial	14 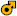 with 4 training sessions * wk, (2 sessions anaerobic power, 2 of aerobic endurance exercise) Age (range): 45–60 y BMI (range): 25–30 kg/m^2^ Body fat (range): 23–30% No withdrawals/lost to follow-up reported	1st 6 wk: 900 mg *TT* (100% steroidal saponins) 2 caps × 300 mg morning on an empty stomach 1 cap × 300 mg bedtime 2nd 6 wk: 1800 mg *TT* (100% steroidal saponins) 4 caps × 300 mg morning on an empty stomach 2 caps × 300 mg bedtime *“Placebo”* caps gelatin 12 wk	Chol LDH-Chol HDL-Chol GH IGF-1 Testosterone	*IG vs. CG* ↓*Chol ↑*HDL-Chol ↓*LDH-Chol ↑*GH ↑*IGF-1 ↑*Testosterone *IG: Change from baseline* ↓*Chol ↑*HDL-Chol ↓*LDH-Chol ↑*GH ↑*IGF-1 ↑*Testosterone	

Abbreviations: ↑ = no significant increase; ↓ = no significant decrease; 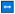 = no significant change. ↑* = significant increase; ↓* = significant decrease; CG = control group; IG = intervention group; wk = weeks; caps = capsules; *TT = Tribulus Terrestris*; DHT = dihydrotestosterone; IGF-1 = Insulin-like growth factor 1; IGFBP-3 = Insulin-like growth factor binding protein 3; GH = growth hormone; CK = creatine kinase; LDH = lactate dehydrogenase; Cr = creatinine; Ua = uric acid; BUN = blood urine nitrogen; RCB = red blood cells; HB = Hemoglobin; HCT = Hematocrit; ESR = erythrocyte sedimentation rate; MCV = mean corpuscular volume; WBC = white blood cells; LYM = lymphocytes MON = monocytes; GRAN = granulocytes; Chol = cholesterol; Tg = triglycerides; LDH-Chol = low-density lipoprotein; 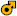 = males; HDL-Chol = high-density lipoprotein; IL-6 = interleukin 6; Hs-CRP = high-sensitivity C-reactive protein; y = years; kg = kilograms; m^2^ = square meters; mg = milligrams.

## Data Availability

All results are shown in the study.
